# Cauda Equina Enhancing Lesion in an HIV-Infected Patient. Case Report and Literature Review.

**DOI:** 10.4084/MJHID.2011.042

**Published:** 2011-10-01

**Authors:** Pasquale De Bonis, Antonella Cingolani, Angelo Pompucci, Tommaso Tartaglione, Luigi M. Larocca, Luciana Teofili

**Affiliations:** 1 Department of Neurosurgery, Catholic University of Rome, Italy; 2 Department of Infectious Diseases, Catholic University of Rome, Italy; 3 Department of Radiology, Catholic University of Rome, Italy; 4 Department of Pathology, Catholic University of Rome, Italy; 5 Department of Hematology, Catholic University of Rome, Italy

## Abstract

We report the case of an HIV-infected young men with neuro-toxoplasmosis localized in the spinal cord. The patient received chemotherapy and immunotherapy for Burkitt lymphoma one year before. At the time of the diagnosis of toxoplasmosis, he was on prophylaxis with trimethoprim and sulfamethoxazole and in complete remission of Burkitt lymphoma. The CD4+ T cell count was 270/μl and the HIV viremia was undetectable. These findings suggest that in this patient, the immunodeficiency promoting the neurologic toxoplasmosis arose more from previous immuno-chemotherapy than from the HIV-infection itself. On the whole, this case highlights that the risk stratification for opportunistic infections of HIV-infected patients should carefully consider their previous medical history and therapies received.

## Introduction

Common anticancer therapies are associated with hematological toxicity and immunological impairment. In particular, patients with lymphomas may cope with a wide spectrum of infectious complications. In this respect, individuals with HIV-related lymphomas represent a subset of patients at high risk for various kinds of infections. Furthermore, several infectious diseases can induce similar symptoms or imaging pictures as lymphomas. As a consequence, the differential diagnosis between the lymphoma and the infection arising from the immunologic deficiency can be sometimes difficult. Here we describe the case of an HIV-positive men with a concomitant Burkitt lymphoma, who developed neuro-toxoplasmosis when he was in complete lymphoma remission, despite the CD4+ T cell count recovery and the trimethoprim/sulfamethoxazole prophylaxis.

## Case report

**Figure 1.**
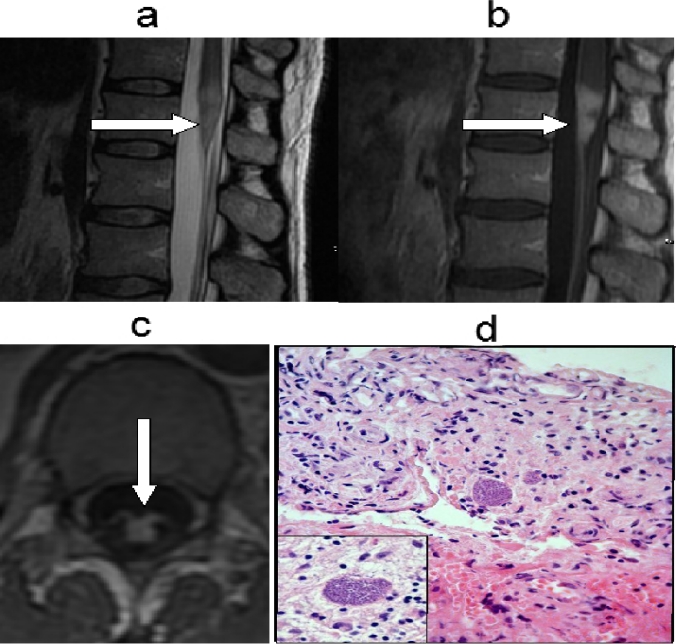
Spine MRI with Gadolinium. The lesion of the conus medullaris involving the proximal portion of cauda equina nerve roots (a), was hyper-intense on T2-weighted images (b) and showed marked contrast-enhancement (c). Histological examination of the conus medullaris biopsy (d) (Hematoxiline eosin staining).

A 44-year-old man was admitted to the infectious disease department of our hospital for fever and abdominal distension. The CT-scan evidenced a 6.5 cm abdominal lymph node mass with peritoneal infiltration. Patient was found HIV-1 infected and the CT driven lymph node biopsy led to the diagnosis of Burkitt lymphoma. At that time, CD4+ T cell count was 55/μl, HIV-RNA copies were 232.000/ml and patient was proven positive for anti-Toxoplasma IgG antibodies. Combined antiviral therapy (cART) with emtricitabine/tenofovir/lopinavir and prophylaxis for Toxoplasmosis/Pneumocystis jiroveci pneumonia and disseminated Mycobacterium Avium Complex infection with trimethoprim/sulfamethoxazole and azithromycin at standard doses were undertaken. In the meanwhile, patient underwent chemotherapy for Burkitt lymphoma, according to the Magrath protocol, consisting of the administration of two regimens: CODOX-M (Cyclophosphamide 800 mg/m^2^ i.v.day 1; Vincristine 1.5 mg/m^2^ i.v. days 1, 8 and 15; Doxorubicin 40 mg/m2 i.v. day 1, Cytarabine 70 mg i.t. days 1 and 3; Cyclophosphamide 200 mg/m^2^ i.v.days 2–5; Methotrexate 1200 mg/m^2^ i.v. over 1 hour and then 240 mg/m^2^ i.v. each hour over 23 hours, day 10; Leucovorin 192 mg/m^2^ i.v. day 11 at hour 36 after methotrexate and then 12 mg/m^2^ i.v. every 6 hours until methotrexate level was not detectable; Methotrexate 12 mg i.t. day 15) and IVAC (Ifosfamide 1500 mg/m^2^ i.v. days 1–5; Mesna 360 mg/m^2^ mixed with ifosfamide i.v. and then 360 mg/m^2^ i.v. 3 hourly for seven doses, Etoposide 60 mg/m^2^ i.v. days 1–5, Cytarabine 2 g/m^2^ i.v. 12 hourly for a total of four doses, days 1–2, Methotrexate 12 mg i.t.day 5)^1^. G-CSF was administered at dose of 5 μg/kg s.c. daily, from day 15 after CODOX-M and from day 7 after IVAC regimen, until neutrophil granulocyte count reached 0,5 × 10^9^/l. Overall, the patient received two alternating CODOX-M/IVAC courses and four Rituximab administrations at 375 μg/m^2^ i.v. dose over a six month period. A total body CT scan performed at the end of therapy, showed normal lymph node, liver and spleen size. The complete remission of the lymphoma was subsequently confirmed by a 18-FDG-PET scan. The CD4+ T cell count arose progressively to normal values and the HIV-RNA copies became persistently undetectable. Four months after the end of the therapy, the patient started to complain progressive weakness of legs, and, afterwards, sudden urinary retention. He was still on prophylaxis with trimethoprim/sulfamethoxazole without interruption; CD4+ T cell count was 270/μl and HIV-RNA was undetectable (< 50 copies/ml). Neurologic examination revealed absent ankle reflexes, reduced strength (3/5) in lower limbs and saddle sensory deficit. The spine MRI with Gadolinium showed a focal lesion of the conus medullaris, involving the proximal portion of the cauda equina nerve roots. The lesion was hyper-intense on T2-weighted images and showed marked contrast-enhancement (Figure 1a–c, **white arrows**). The patient was then admitted to our department: the brain MRI demonstrated an additional lesion in the deep right frontal region (0.5 cm), with signal alterations similar to that of the spinal cord. The cerebral spinal fluid (CSF) examination showed a slight increase of protein and cell concentrations (199 mg/dl and 56/mm^3^, respectively), with any evidence of immature lymphoid cells. The flow cytometry analysis of CSF samples revealed that cells were CD19 positive B lymphocytes, without κ or λ light chain restriction. PCR assays for Mycobacterium tuberculosis-DNA, Epstein Barr virus (EBV)-DNA and cytomegalovirus-DNA on CSF were all negative. The search for Cryptococcus antigen was negative, as well. Whereas both brain and spinal lesions were highly suggestive for lymphoma localizations, the total body CT scan showed no liver, spleen or lymph node enlargement consistent with the systemic relapse of the Burkitt lymphoma. Indeed, in the suspicion of an isolated CNS relapse, the patient underwent open biopsy of the conus medullaris lesion. The histological examination revealed numerous Toxoplasma tachyzoites surrounded by inflammatory cells (Figure 1d). Actually, the patient referred that he owned a cat who lived with him. Indeed, the proper treatment with pyrimethamine and sulfadiazine was undertook with marked improvement of neurologic conditions in few weeks. The patient showed the full neurologic recovery and the MRI scan performed after 4 months demonstrated the definite disappearance of both spinal and brain lesions.

## Discussion

This case underscores how the detection of spinal cord enhancing lesion in AIDS patients represents a diagnostic challenge. In particular, in this patient, the pre-existing diagnosis of Burkitt lymphoma and the unusual inaugural spinal cord localization of toxoplasmosis undoubtedly hampered the diagnosis of neuro-toxoplasmosis. Actually, especially in HIV-infected people, Burkitt lymphoma exhibits a marked CNS tropism,^1^ while spinal cord toxoplasmosis has been rarely reported. Vyas and Ebright described fourteen cases of spinal cord toxoplasmosis in AIDS patients; afterwards, one additional case has been recently reported.^4^ Eleven out of these 15 patients showed also brain lesions: importantly, the mortality rate in this series of patients is 40 % (6 out of 15 patients).^3^ However, in our opinion, the case here reported is remarkable because of neurologic toxoplasmosis occurred in spite of the proper prophylaxis and with a CD4+ T cell count higher than 200/μl. Actually, toxoplasma causes encephalitis mainly in severely immune-compromised patients, or in those not receiving primary prophylaxis.^3^ In contrast, immuno-recovered patients are considered at low risk for this complication, so that, in this subset of patients, the interruption of prophylaxis has been recently proposed.^6^ Interestingly, a case of solitary spinal cord toxoplasmosis has been reported by Straathof in an HIV-negative patient undergoing autologous stem-cell transplantation for multiple myeloma.^7^ Actually, in the setting of hematological patients receiving high dose chemotherapy, cerebral toxoplasmosis constitutes one of the most frequent neurologic complication and is associated with a high mortality rate.^6^ Similarly, our patient received intensive chemotherapy and immunotherapy for Burkitt lymphoma, probably resulting in the persistent dysfunction of cell-mediated immunity despite the adequate lymphocyte count.

The cART dramatically reduced the incidence of CNS opportunistic infections in HIV-infected people; in addition, its association with intensive chemotherapies significantly improved the prognosis of patients with HIV-related lymphomas. Nonetheless, chemotherapy and immunotherapy related toxicities resulted in a wide spectrum of infectious complications. Importantly, this case highlights how the CD4+ T cell count and the HIV RNA are not suitable tools to predict the risk of opportunistic infections in HIV-infected people with lymphomas.
